# Non-Equilibrium Thermodynamic Analysis of Transport Properties in the Nanofiltration of Ionic Liquid-Water Solutions

**DOI:** 10.3390/molecules14051781

**Published:** 2009-05-11

**Authors:** Bo Wu, Yu M. Zhang, Hua P. Wang

**Affiliations:** State Key Laboratory for Modification of Chemical Fiber and Polymer Materials, Donghua University, Shanghai 201620, China

**Keywords:** Ionic liquid, nanofiltration, irreversible thermodynamics, transport coefficients, rejection

## Abstract

The nanofiltration of aqueous solutions of the ionic liquids (ILs) 1-butyl-3-methylimidazolium tetrafluoroborate ([Bmim]BF_4_), and 1-butyl-3-methylimidazolium bromide ([Bmim]Br) with a polyamide nanofiltration membrane was investigated. The practical transport coefficients, including hydrodynamic permeability (*L*_p_), reflection (*σ*) and solute permeability (*ω*) were calculated in terms of a non-equilibrium thermodynamics approach. It was found that *L*_p_ and *σ* diminished as the concentration of the IL solutions increased. These characteristics are similar to those observed in inorganic electrolyte-water systems. In addition, it was shown that the rejection and volume flux for both ionic liquid solutions rose with feed pressure, while it decreased with feed concentration. The maximum rejection efficiencies for [Bmim]Br and [Bmim]BF_4_ are 67 % and 60 %, respectively, on our experimental scale. All the data suggests that a highly efficient process for IL separation could be developed when the operating conditions are optimized further.

## 1. Introduction

Ionic liquids (ILs) are a class of compounds reputed as green solvents that is being extensively studied nowadays. Due to their lack of volatility, these liquids are generally thought to be easily recoverable from common organic solvents through distillation [1], but large scale industrial implementation of distillations consumes a great amount of energy, therefore it may not be practical to separate hydrophilic ILs from water solutions through direct evaporation or distillation, especially if the IL is the minor component in the mixture [2].

Membrane separation techniques have been successfully applied in the fields of concentration, separation and desalination, concomitant with development of some suitable mathematical models. For example, a non-equilibrium thermodynamic model for the separation of inorganic salts has been extensively developed, based on the fact that all membrane permeation and separation processes are non-equilibrium processes [3,4,5,6,7].

Recently, Gan *et al.* found that direct filtration of the pure ILs [C_10_ min]NTf_2_ and [N_8881_]NTf_2_ through Nuclepore™ microfiltration membranes yielded very low permeation rates, which can be easily enhanced by mixing with a small volumetric proportion of methanol or ethanol [8]. Based on these findings, Livingston *et al.* and Kröckel *et al.* separated ILs from reaction products using the nanofiltration technique [9,10,11], indicating that it is possible to separate hydrophilic ILs from their aqueous solutions with nanofiltration membranes. Fernández *et al*. [12] recently reported recovery of the IL 1-octyl-3-methyl-imidazolium chloride ([C_8_mim]Cl) from waste water through aggregation control by addition of FeCl_3_. Their experiments are based on the fact that [C_8_mim]^+^ should show inherent amphiphilicity and exhibit aggregation behaviour analogous to the properties of surfactants, but no detailed information about the mode of operation, the efficiency and the technical potential of such a process has been reported either in the patent literature or in any other publication. In our very recent paper [13], we proposed that if effective recovery of ILs from their dilute aqueous solutions is desired, these solutions should be concentrated using nanofiltration membrane processes, so it is necessary to study the transport properties of hydrophilic ILs in membrane processes in order to better understand the separation of ILs with nanofiltration membranes. 

In this work, pressure-driven filtration experiments were performed, using dilute aqueous solutions of 1-butyl-3-methylimidazolium tetrafluoroborate ([Bmim]BF_4_), and 1-butyl-3-methylimidazolium bromide ([Bmim]Br), as the feed. The first objective of this investigation was to obtain nanofiltration data for the ILs-polyamide membrane system and demonstrate the potential of this nanofiltration technique for concentrating ILs. A further goal is to analyze the results of the nanofiltration experiments by the non-equilibrium thermodynamics method to obtain the practical transport coefficients. Finally, we compared the practical transport coefficients for this ILs-water system with those of an inorganic electrolyte-water system.

## 2. Theory

The non-equilibrium thermodynamics mass transport model was previously described in detail for electrolyte solutions [5]. By considering the membrane as a black-box, the practical transport coefficients of a binary solution, including the hydrodynamic permeability coefficient (*L*_p_); the reflection coefficient (*σ*); and the solute permeability coefficient (*ω*) were derived from irreversible thermodynamics. These parameters can be evaluated by measuring the volume flux (*J*_v_) and the rejection of the solute (*r*) as a function of pressure and utilizing equation (1):

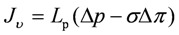
(1)

Following Kedem [3], a linear relationship for the reciprocal solute rejection, 1/*r*, as a function of the reciprocal volume flux, 1/*J*_v_, is obtained:

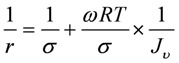
(2)
where *R* is the gas constant, *T* the temperature, Δ*p* the pressure and Δ*π* the osmotic pressure difference across the membrane. 

The osmotic pressures (*π*) of the feed and permeate solutions, were calculated by:

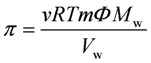
(3)
where *v* is the number of ions formed from the electrolyte, *m* its molality, *M*_w_ the molar mass of water, *V*_w_ the molar volume of water. *Φ* is the osmotic coefficient of the solution and can be calculated from Pitzer’s theory [14,15] using the equation for a 1-1 electrolyte:

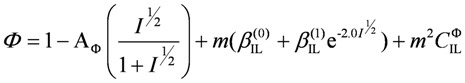
(4)
where *I* is the ionic strength. 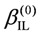, 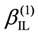 and 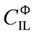 are characteristic parameters of ILs available in the literature [16]. A_Φ_ is the Debye-Hückel coefficient for the osmotic function, with values given by Bradley and Pitzer [17]. In the calculation of the osmotic coefficient (*Φ*), molarity must be converted to molality.

More flows can occur in an electrolyte solution than in a non-electrolyte solution for the same number of components, in the case of a two component solution of a monovalent electrolyte in the absence of an electric current:

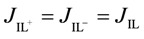
(5)
where 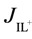, 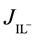
and 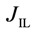 are the fluxes of cation-ILs, anion-ILs and ILs, respectively. The problem is reduced to only two fluxes, 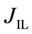 and the water flux (*J*_w_), and the definitions of the transport coefficients (*L*_p_, *σ* and *ω*) also retain their validity in the case of electrolyte solutions. 

## 3. Experimental Section

[Bmim]BF_4_ and [Bmim]Br were prepared based on the reported procedures [18]. Aqueous solutions of [Bmim]BF_4_ and [Bmim]Br of various concentrations were freshly prepared. The viscosities of the samples, the values of which are listed in Table 1, were measured in triplicate at the operating temperature with an Ostwald viscometer. All nanofiltration experiments were carried out in experimental membrane separation units over the pressure range 0.1-0.5 MPa, maintaining a constant temperature of 318 ± 1 K. TFC^®^ 3838 SR^®^3 membrane modules (polyamide, supplied by M Koch Membrane Sytems), supported by a stainless steel cylinder, were used in all nanofiltration experiments.

The volume flux, *J*_v_, (m/s) and rejection, *r*, were measured. The volume flux was determined by measuring the volume of permeate collected in a time interval. The concentrations of ILs in the permeate and retentate were determined with a UV spectrophotometer (UV–visible 1200 spectrometer) at a wavelength of 212 nm. Rejection was calculated by equation (6):

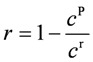
(6)
where *c*^p^ and *c*^r^ are the ILs concentrations in the permeate and retentate.

**Table 1:** Viscosities and Practical transport coefficients (*L*_P_ × 10^7^ m/s MPa, *σ*, *ω* × 10^4^ mol/m^2^s MPa) fornanofiltration of aqueous solutions of Ils.

Parameter	[Bmim]BF_4 _*m* (mol/kg)				[Bmim]Br *m* (mol/kg)			
	0.044	0.221	0.442	0.663	0.045	0.228	0.456	0.684
***L*_P_ (± 0.01)**	8.97	4.34	3.75	3.05	8.73	5.50	5.21	4.83
***σ* (± 0.01)**	0.79	0.74	0.45	0.40	0.83	0.70	0.54	0.49
***ω* (± 0.02)**	5.21	7.67	3.82	4.46	4.23	5.04	3.62	6.04
***η* (± 0.01)**	0.90	0.95	1.05	1.11	0.92	0.98	1.08	1.14

## 4. Results and Discussion

### 4.1. Nanofiltration

**Figure 1:**
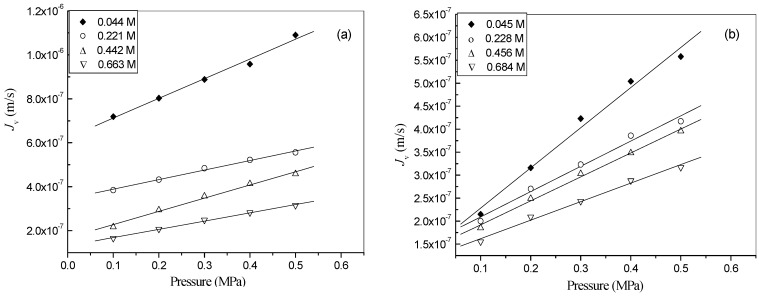
Dependence of volume flux on pressure for (a) [Bmim]BF_4_ solution; (b) [Bmim]Br solution.

The volume flux (*J*_v_) and rejection (*r*) were obtained from the nanofiltration experiments. As can be seen from Figure 1, the permeate flux of both solutions increases linearly with pressure, which indicates that there is insignificant concentration polarization in the membrane cell [7]. 

**Figure 2:**
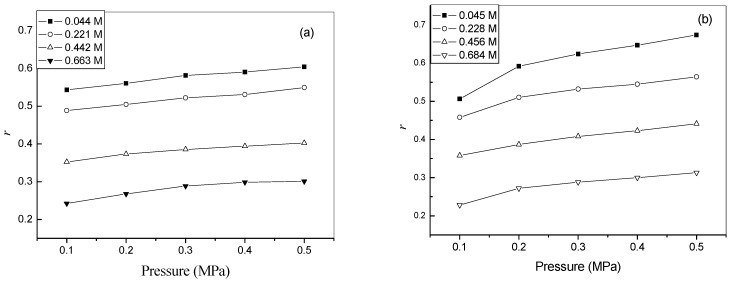
Dependence of rejection on pressure for (a) [Bmim]BF_4_ solution; (b) [Bmim]Br solution.

As expected, *J*_v_ decreases with an increase in concentration of the IL solutions. Interestingly, the values of *J*_v_ for [Bmim]Br solutions are slightly smaller than that for [Bmim]BF_4_ solutions. This is due to the fact the viscosities of the former are slightly higher than those of the latter, as can be observed from Table 1. This reason can be also used to explained why the rejection of [Bmim]Br (0.67) is higher than that of [Bmim]BF_4_ (0.60), which is shown in Figure 2. Additionally, it can also be observed that the rejections of both ILs increase linearly with applied pressure, while they decrease with the rise of feed concentration.

### 4.2. Transport coefficients from nanofiltration experiments

Based on the experimental data, the practical transport coefficients (*L*_p_, *σ* and *ω*) were determined using the procedure described elsewhere [3,5]. The reflection coefficients, *σ*, were obtained the linear relationship between reciprocal rejection and reciprocal flux (Eq. 1) from the intersection of the corresponding regression line with the ordinate. The solute permeability coefficients, *ω* (m/s), were obtained from the slope of the resulting regression line. The hydrodynamic permeability coefficients, *L*_p_(m/s MPa), were obtained from linear relationships between *J*_v_ and (Δ*p*-*σ*Δ*π*) (Eq. 2) with the *σ* values evaluated using the Eq.1. In the evaluation of *L*_p_, Δ*π* was calculated with the Pitzer relationship for electrolyte solutions (Eqs. (3) and (4)) as in the literature. The values of the parameters required for the calculation of *Φ* were obtained from the literature: A_Φ_ = 0.4065, 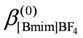 = 1.0369 and 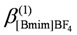 = ‑3.8953 for [Bmim]BF_4_; 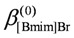 = 0.1435, 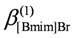 = -1.498 for [Bmim]Br at 318 K [15,16]. Since the molalities of both ILs solutions are less than 1.5 mol·kg^-1^, the third virial coefficient, 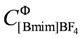, is very small and thus neglected in the calculation. The results were listed in Table 1.

As can be seen from Table 1, surprisingly, the solute permeability coefficient (*ω*) was not a function of concentration. This result is similar to that obtained by Spencer and Narsaian [4,19]. The reason is not clear yet and is under further study. The hydrodynamic permeability (*L*_p_) and reflection coefficient (*σ*) decreased as concentration rose for both ILs, which is consistent with the results in the literature [5,7]. An additional point to note is that the values of *σ* for [Bmim]Br are higher than that for [Bmim]BF_4_, although the size of Br^-^ (196 pm) is smaller than that of BF_4_^-^ (232 pm) [20]. This may be attributable to the aforementioned viscosity difference. Another reason might be related to ion hydration, which will be discussed in the following section. 

Furthermore, one noticeable feature is that the values of *σ* obtained in this work are lower than those of the NaCl-water systems [5], although the sizes of [Bmim]^+^ (331 pm), BF_4_^-^ (232 pm) and Br^-^ (196 pm) are all larger than that of Na^+^ (102 pm), Cl^-^ (181 pm) [20]. This behavior is attributable to the difference of hydrations of ions. Hydrated ions have a definite geometric structure. When the hydrated ion is forced under pressure through the pore of the membrane, the hydrated ion's geometric structure can be crushed out of shape, that is, the water molecules around the ion have been partially dehydrated. The stronger the hydrated structure, the more difficult to be crush out of shape under the same conditions. The hydration of NaCl is stronger than that of both ILs; the respective hydration numbers are 5.5 for NaCl, 2.7 for [Bmim]BF_4_ and 2.9 for [Bmim]Br [20]. Therefore, ILs go through the membrane easier, and this is another reason why the values of *σ* for [Bmim]BF_4_ are smaller than those for [Bmim]Br. More importantly, this behavior may indicate that there is almost no aggregation behavior in aqueous [Bmim]BF_4_ or [Bmim]Br solution when the molarity is less than 0.66 mol/L, which is consistent with the critical aggregation concentration of [Bmim]BF_4_ (CAC = 0.81 mol/L) of Bowers [21] and [Bmim]Br (CAC = 0.80 ± 0.10mol/L) given by Goodchild [22]. On this basis, one might question what results can be obtained if the concentration of the solution were superior to the CAC, even if ILs with long alkyl chain, like [Omim]Cl mentioned above, were used in nanofiltration. These facts need further research.

This work is just an exploratory study on the possibility of utilizing nanofiltration membrane to separate ILs from aqueous solutions, and the separation efficiency will be affirmed through optimizing varied conditions. Nevertheless, as a contribution to a better understanding of IL separation and the development of an efficient process, the separation of IL using nanofiltration technique was studied as an instructive example.

## 5. Conclusions

An irreversible thermodynamic model was successfully used to estimate the practical membrane transport coefficients, *L*_p_, *σ* and *ω*. The relations of *L*_p_ and *σ* with concentration of solute are similar to but lower than those obtained for inorganic electrolyte-water solutions. Although the maximum rejection efficiency of ILs is only 60 % for [Bmim]BF_4_ and 67 % for [Bmim]Br, it can be concluded from transport coefficients that nanofiltration may be most promising way for concentrating dilute aqueous solutions of ILs, if the nanofiltration conditions can be improved.
